# Equity in the geographical distribution of general practitioners in Iran

**DOI:** 10.1371/journal.pgph.0003609

**Published:** 2024-09-24

**Authors:** Farahnaz Khajehnasiri, Ali Mohammad Mosadeghrad, Mohammad Hossein Alibeiginejad

**Affiliations:** 1 Department of Community Medicine, School of Medicine, Tehran University of Medical Sciences, Tehran, Iran; 2 Department of Health Management, Policy and Economics, School of Public Health, Tehran University of Medical Sciences, Tehran, Iran; 3 Medical Student, School of Medicine, Tehran University of Medical Sciences, Tehran, Iran; Alexandria University Faculty of Dentistry, EGYPT

## Abstract

**Background:**

The equitable distribution of workforce is imperative for achieving public health goals. This study was conducted to evaluate the equity of the distribution of general practitioners (GPs) in Iran.

**Methods:**

In this descriptive and cross-sectional study, data from the Information System of Benefits for Iranians and the Iranian Statistics Center were utilized. The study population encompasses all GPs operating in Iran in 2022. To assess equity in the geographical distribution of physicians, the Gini coefficient and Lorenz curve were calculated using Excel software. Additionally, ArcGIS software was used to create a distribution map.

**Results:**

The population of Iran and active GPs in 2022 were 85,874,000 and 67,852 respectively, resulting in an average of 7.9 active GPs per 10,000 people. The provinces of Yazd, Tehran, Mazandaran, and Fars exhibited the highest concentration of active GPs per capita, collectively representing more than 28% of the population and 37% of the active GPs. Fifty percent of the active GPs were women. The Gini coefficients for the provincial and intercity distributions of GPs were 0.08 and 0.2, respectively.

**Conclusion:**

The distribution of GPs among provinces and cities was deemed fair and relatively fair, respectively. Iran has a lower ratio of GPs per capita than the European nations. Policymakers should pay more attention to the implementation of appropriate policies with the aim of increasing equity in the geographical distribution of GPs in the country.

## Introduction

The health system constitutes a network of organizations, groups, and individuals involved in creating resources, financing, stewardship, and delivering healthcare services within both the governmental and nongovernmental sectors. The overall goals of a health system encompass health improvement, responsiveness, and financial risk protection. The intermediate objectives of the health system involve the achievement of overall goals, including access, safety, quality, efficiency, and coverage. The World Health Organization has outlined the prerequisites for realizing these goals within the framework of six building blocks, namely, leadership and governance, financing, human resources, access to essential medicines, health information systems, and service delivery [1].

In the framework of the six building blocks outlined by the WHO, human resources for health (HRH) play a crucial role in achieving universal health coverage and realizing the Sustainable Development Goals (SDGs) while promoting social equity [2]. Equitable allocation of specialized human resources to the population is a requisite condition for achieving UHC [3]. On the one hand, the responsibility of the human workforce lies in utilizing health technologies to deliver services with the aim of promoting health status; on the other hand, a considerable portion of the total current expenditure within the healthcare sector is allocated to the expenses associated with training, recruiting, and retaining the human workforce [4].

Facilitating effective healthcare interventions requires a diverse array of skilled professionals within the health system. The management of human resources is of crucial importance in enabling the delivery of efficient and effective services in the health care system [5]. Among the human resources available within the health system, physicians assume a significant role. The disparity between the existing number of physicians and the current and future demands of the health system, coupled with their uneven distribution across the nation, creates substantial impediments to the utilization of other facilities and resources within the health system [6]. According to existing studies, the enhancement of healthcare service quality and the amelioration of community health status hinge not only on the quantity and availability of physicians but also on other factors, inclusive of the equitable distribution of these resources across different regions of the country [7]. The World Health Organization has also identified equity and access as pivotal goals of global health systems [8].

Several definitions of equity have been proposed [9], one of which was formulated by Whitehead in 1992: Inequity in health is a category of avoidable and unnecessary health inequalities that are created unfairly [10]. Concerning distribution, equity can be categorized as horizontal or vertical equity. Horizontal equity (equality) entails treating individuals with identical needs equally. However, in the realm of the health system, individuals have diverse needs, necessitating the consideration of vertical equity. Vertical equity involves catering to individuals in consonance with their specific needs, ensuring that individuals with differing requirements receive healthcare services commensurate with their needs. In the health system, the scope of equity can be delineated across three primary areas: financing, healthcare provision (access), and the populace’s health status. Hence, health equity signifies access to healthcare services based on necessity, payment for services according to financial capability, and the attainment of an acceptable level of health, ensuring uniform health status for all members of society regardless of age, gender, race, or socioeconomic standing [11]. A fundamental challenge in the realm of public health and social disparities, especially in deprived areas, is the uneven distribution of physicians in relation to the populace [12], which has led to compromised access to healthcare services in Iran [13].

Iran is the seventeenth largest country in the world with an area of 1.648 million square kilometers and about 86 million people of which about 61 million live in urban areas [14]. Iran’s healthcare system is overseen by the Ministry of Health and Medical Education (MoHME). This ministry is responsible for providing health services, medical education, research, and health policy formulation [15]. Iran has a robust medical education system with numerous medical schools across the country. As of the latest data, there are 67 medical universities across 31 provinces in Iran [16]. The healthcare system in Iran is predominantly public, with the government playing a key role in funding and providing healthcare services [17]. In Iran, health services are funded through a combination of government expenditure, insurance schemes, and out-of-pocket payments by individuals. The government heavily subsidizes healthcare, aiming to make services affordable and accessible [18]. Basic health insurance is mandatory in Iran. According to the law, the government must insure those who cannot afford to pay the premium for free. So large portion of the Iranian population is covered by health insurance, with over 92% insured under various public and semi-public schemes [16]. Iran has made significant strides towards achieving Universal Health Coverage (UHC). The Health Transformation Plan (HTP), launched in 2014, aimed to enhance access to healthcare, improve quality, and reduce out-of-pocket expenses. The government continues to work towards comprehensive coverage, although disparities in healthcare access remain between urban and rural areas [18].

Previous studies conducted in recent years in Iran have underscored the inequitable distribution of physicians among the country’s provinces [19–21]. Nevertheless, policymakers encounter constraints in utilizing these studies, such as the passage of time since the study year, single-province focus, calculation of distribution only among public sector physicians, and amalgamation of active and inactive physicians in the calculation [10,20]. Hence, the current research was undertaken with the objective of assessing equity in the geographical distribution of general practitioners (GPs) in Iran (within both the governmental and nongovernmental sectors). The findings derived from this research will furnish policymakers and health system managers with valuable insights for the judicious management of medical workforce across the country.

## Methods

The current cross-sectional study employed a descriptive-analytical approach. The statistical population comprises all active GPs in Iran in 2022. The required data was received by sending a written request on 05/15/2023. The data pertaining to the frequency of active GPs categorized by age, sex, province, and city of practice in 2022 were sourced from the Information System of Benefits of Iranians (Ministry of Cooperation, Labor, and Social Welfare). Additionally, data pertaining to the population of each province and city in 2022 were obtained from the Iranian Statistics Center. Subsequently, the data were analyzed using Excel 2019 software, and the Gini coefficient for the distribution of GPs was computed. Finally, a physician distribution map was generated utilizing ArcGIS Pro software.

ArcGIS Pro software was used to map the geographical distribution of GPs across provinces and cities. A gradient color scheme, ranging from red to green, was applied to indicate GPs density, with red representing lower densities and green representing higher densities. This method allowed for a visually appealing representation of the data, facilitating better understanding of the distribution patterns and aiding in the identification of regions with potential healthcare service disparities [22].

The allocation of medical professionals was evaluated using the established methodologies of the Gini coefficient and the Lorenz curve. The Lorenz curve, conceived by Max Lorenz in 1905, is utilized to assess the level of inequality in individuals’ income within a society. Comparing the distribution of a variable with its ideal distribution, the curve portrays the cumulative percentage of the population on the X-axis and the cumulative percentage of the researched variable on the Y-axis. In a scenario of perfect equity, the distribution line would coincide with the 45-degree line on the graph. As the variable distribution curve moves away from this line, the level of inequality increases. In a situation of complete unfairness, this line aligns with the X-axis. To measure this inequality, the Gini coefficient, developed by Corrado Gini in 1910, was employed. The Gini coefficient serves as an appropriate indicator for assessing equity in the allocation of resources within the healthcare system [23].

**Fig 1:**
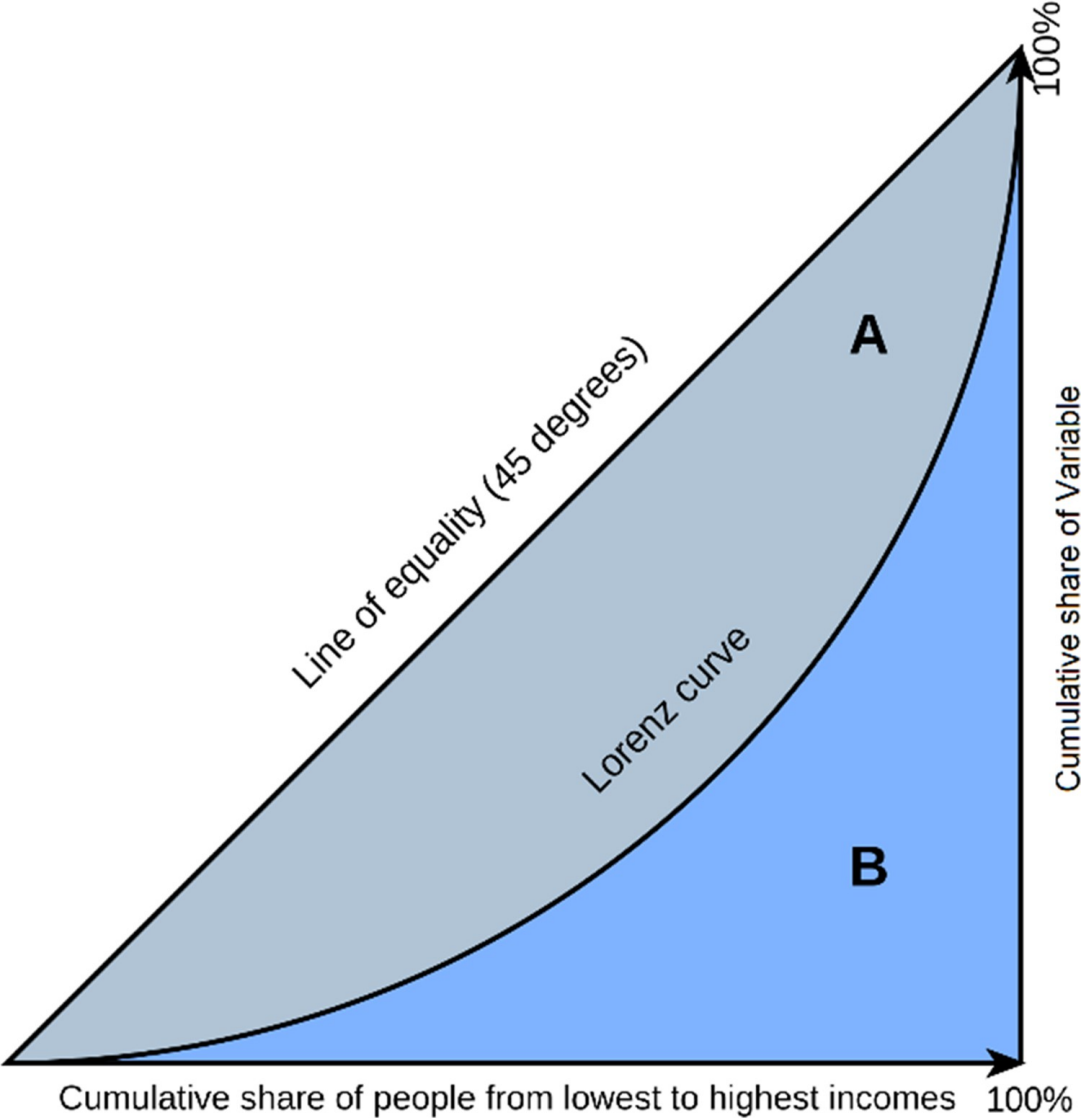
Lorenz curve.

The Gini coefficient is considered a suitable indicator for evaluating the fairness of the allocation of resources within the healthcare system [24]. This coefficient represents the ratio of the area between the Lorenz curve and the 45° line to the total area under the 45° line (Fig 1). In this figure A is the area between the Lorenz curve and the line of equality (the 45-degree line) which represents the degree of inequality in the distribution of the variable and B is the area under the Lorenz curve. So the Gini coefficient is A/(A+B).

The Gini coefficient can also be calculated using the following formula:

$$Gini=\sum_{i=1}X_{i}Y_{i+1}-\sum_{i=1}X_{i+1}Y_{i}$$

In this formula, "X" denotes the cumulative percentage of the population, and "Y" represents the cumulative percentage of the studied variable. The resulting numerical coefficient falls within the range of zero to one, where a Gini index approaching zero signifies a more equitable distribution of the variable within the studied society. A Gini coefficient below 0.2 indicates complete fairness in distribution, while a Gini coefficient ranging from 0.2 to 0.3 suggests a relatively fair distribution. Moreover, a Gini coefficient spanning from 0.3 to 0.4 indicates relative inequality in distribution, and a Gini coefficient of 0.4 to 0.5 signifies significant inequality in distribution, whereas a Gini coefficient exceeding 0.5 connotes complete inequality in distribution [25].

For the calculation of the Lorenz curve and the Gini coefficient, units of analysis were examined at three distinct levels: interprovincial, intercity and intraprovincial levels. At the interprovincial level, the number of provinces was 31. At the intercity level, the number of cities was 429. And at the intraprovincial level it was different for each province from 1 to 29. These units were used as the basis for statistical calculations and the analysis of the distribution of GPs.

This research was ethically approved by the Ethics Committee of Tehran University of Medical Sciences with the code IR.TUMS.MEDICINE.REC.1400.022. All methods were conducted in accordance with relevant guidelines and regulations. All the data was obtained with permission from relevant organizations.

## Results

The data derived from this study revealed that in the year 2022, 67,852 active GPs practiced medicine across all 31 provinces of the country, 50% of whom were female. The overall density of active GPs in Iran was 7.9 per ten thousand people. Table 1 displays both the total count and the per capita count of active GPs per ten thousand people, categorized by province of practice.

**Table 1:** Number and per capita of active GPs in 2022 by location of practice (per capita per 10,000 people).

Provinces	Life expectancy [26]	The centers of the provinces					Other cities				
		Population (thousands)		General practitioner			Population (thousands)		General practitioner		
		Number	percentage	Number	percentage	per capita	Number	percentage	Number	percentage	per capita
Alborz	78.6	1,895	5.00%	1,457	3.40%	7.7	1,111	2.30%	453	1.80%	4.1
Ardabil	73	652	1.70%	571	1.30%	8.8	669	1.40%	342	1.40%	5.1
Bushehr	74.8	338	0.90%	277	0.60%	8.2	953	2.00%	452	1.80%	4.7
Chaharmahal and Bakhtiari	77	350	0.90%	429	1.00%	12.3	656	1.40%	402	1.60%	6.1
East Azerbaijan	73.1	1,895	5.00%	2,247	5.20%	11.9	2,217	4.60%	849	3.40%	3.8
Fars	76.8	2,046	5.40%	2,944	6.90%	14.4	3,088	6.40%	1,911	7.70%	6.2
Gilan	73.3	1,001	2.60%	1,189	2.80%	11.9	1,586	3.30%	1,219	4.90%	7.7
Golestan	74	524	1.40%	622	1.50%	11.9	1,498	3.10%	723	2.90%	4.8
Hamadan	75	717	1.90%	791	1.80%	11	1,078	2.20%	485	1.90%	4.5
Hormozgan	75.6	784	2.10%	582	1.40%	7.4	1,237	2.60%	531	2.10%	4.3
Ilam	76.6	257	0.70%	235	0.50%	9.2	353	0.70%	215	0.90%	6.1
Isfahan	78.3	2,333	6.20%	2,754	6.40%	11.8	3,104	6.50%	2,292	9.20%	7.4
Kerman	76.4	813	2.10%	1,189	2.80%	14.6	2,610	5.40%	1,352	5.40%	5.2
Kermanshah	75.1	1,168	3.10%	1,138	2.70%	9.7	850	1.80%	440	1.80%	5.2
Khuzestan	74.3	1,431	3.80%	1,660	3.90%	11.6	3,600	7.50%	1,598	6.40%	4.4
Kohgiluyeh and Boyer-Ahmad	76.1	342	0.90%	356	0.80%	10.4	430	0.90%	243	1.00%	5.7
Kurdistan	74.2	542	1.40%	445	1.00%	8.2	1,166	2.40%	502	2.00%	4.3
Lorestan	76.9	536	1.40%	435	1.00%	8.1	1,281	2.70%	529	2.10%	4.1
Markazi	77.2	618	1.60%	549	1.30%	8.9	880	1.80%	505	2.00%	5.7
Mazandaran	76.4	534	1.40%	915	2.10%	17.1	2,906	6.10%	2,344	9.40%	8.1
North Khorasan	74.9	359	0.90%	247	0.60%	6.9	553	1.20%	285	1.10%	5.1
Qazvin	75.8	639	1.70%	631	1.50%	9.9	725	1.50%	318	1.30%	4.4
Qom	75.5	1,447	3.80%	898	2.10%	6.2	-	-	-	-	-
Razavi Khorasan	75.3	3,715	9.80%	3,128	7.30%	8.4	3,356	7.00%	1,703	6.80%	5.1
Semnan	76.4	232	0.60%	245	0.60%	10.6	562	1.20%	397	1.60%	7.1
Sistan and Baluchestan	72.9	807	2.10%	625	1.50%	7.7	2,371	4.90%	741	3.00%	3.1
South Khorasan	73.8	292	0.80%	326	0.80%	11.2	555	1.20%	305	1.20%	5.5
Tehran	80.1	9,204	24.30%	13,319	31.10%	14.5	5,085	10.60%	2,261	9.10%	4.4
West Azerbaijan	72.6	1,148	3.00%	1,035	2.40%	9	2,368	4.90%	861	3.40%	3.6
Yazd	78.5	742	2.00%	1,054	2.50%	14.2	538	1.10%	435	1.70%	8.1
Zanjan	76	565	1.50%	580	1.40%	10.3	562	1.20%	286	1.10%	5.1
**Total**	**75.9**	**37,926**	**100%**	**42,873**	**100%**	**11.3**	**47,948**	**100%**	**24,979**	**100%**	**5.2**

As shown in Table 1, Yazd Province was the top-ranked province on the list, with a total of 11.6 GPs per ten thousand people. Tehran Province followed this, with 10.9 per capita, and Mazandaran and Fars provinces, with 9.5 per capita. Conversely, the provinces of Sistan and Baluchestan had the lowest density of GPs, with 4.3 per capita; Lorestan, with 5.3 per capita; West Azerbaijan, with 5.4 per capita; and Hormozgan, with 5.5 per capita. The provincial capitals of Mazandaran, Kerman, Tehran, and Fars exhibited the highest density of active GPs per ten thousand people, recording 17.1, 14.6, 14.5, and 14.4 per capita, respectively. Conversely, the provincial centers of Qom, North Khorasan, and Hormozgan had the lowest per capita counts among all the provincial centers, with 6.2, 6.9, and 7.4 active GPs per ten thousand people, respectively.

A comparison of the cities within each province (excluding the provincial capital) revealed that the cities of Yazd, Mazandaran, and Gilan boasted the highest density of active GPs per ten thousand people, with 8.1, 8.1, and 7.7 practitioners, respectively. On the other hand, the cities of Sistan and Baluchestan, West Azerbaijan, and East Azerbaijan reported the lowest per capita counts, documenting 3.1, 3.6, and 3.8 active GPs per ten thousand people, respectively.

**Fig 2:**
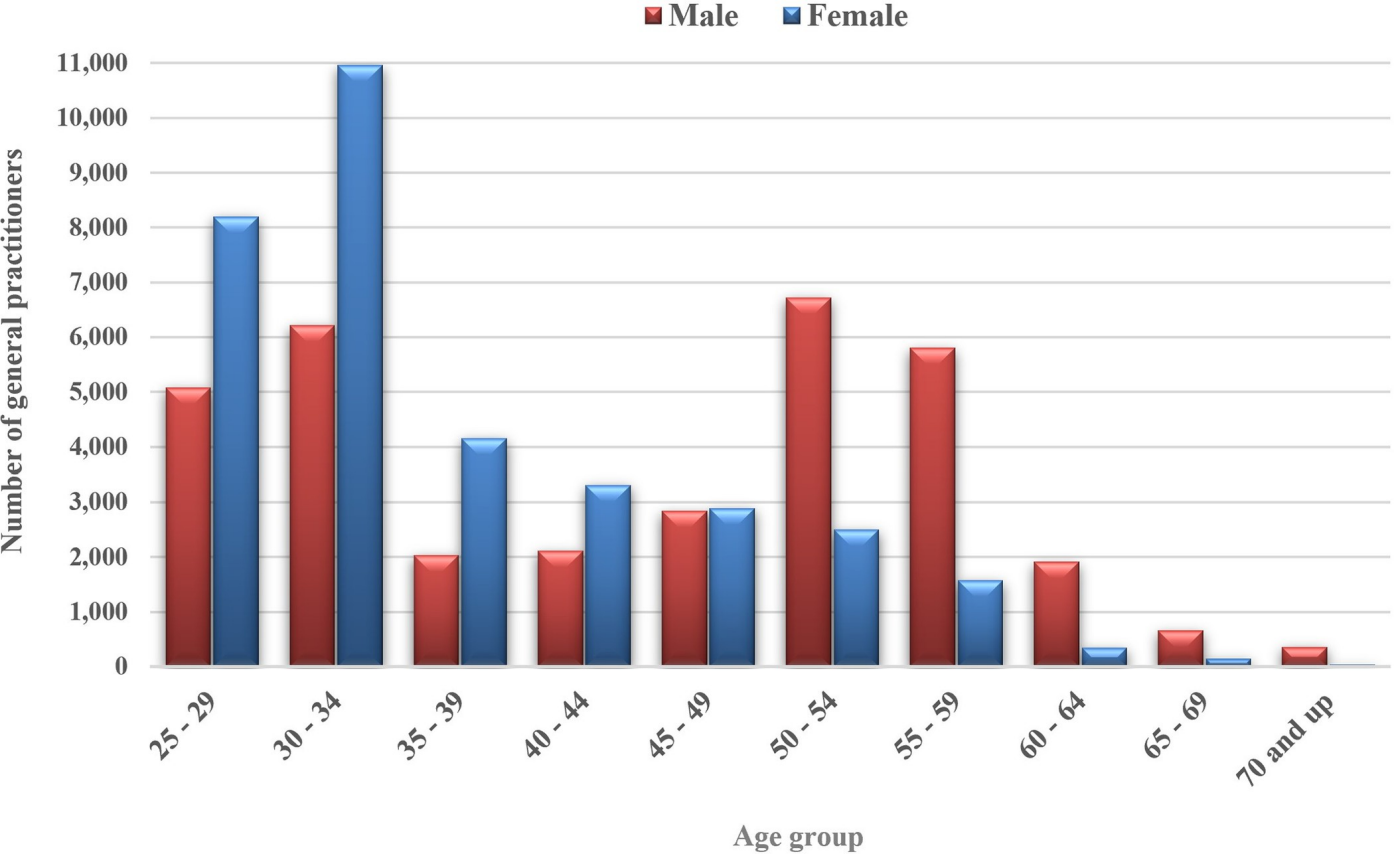
The number of GPs in each age group by gender.

**Fig 3:**
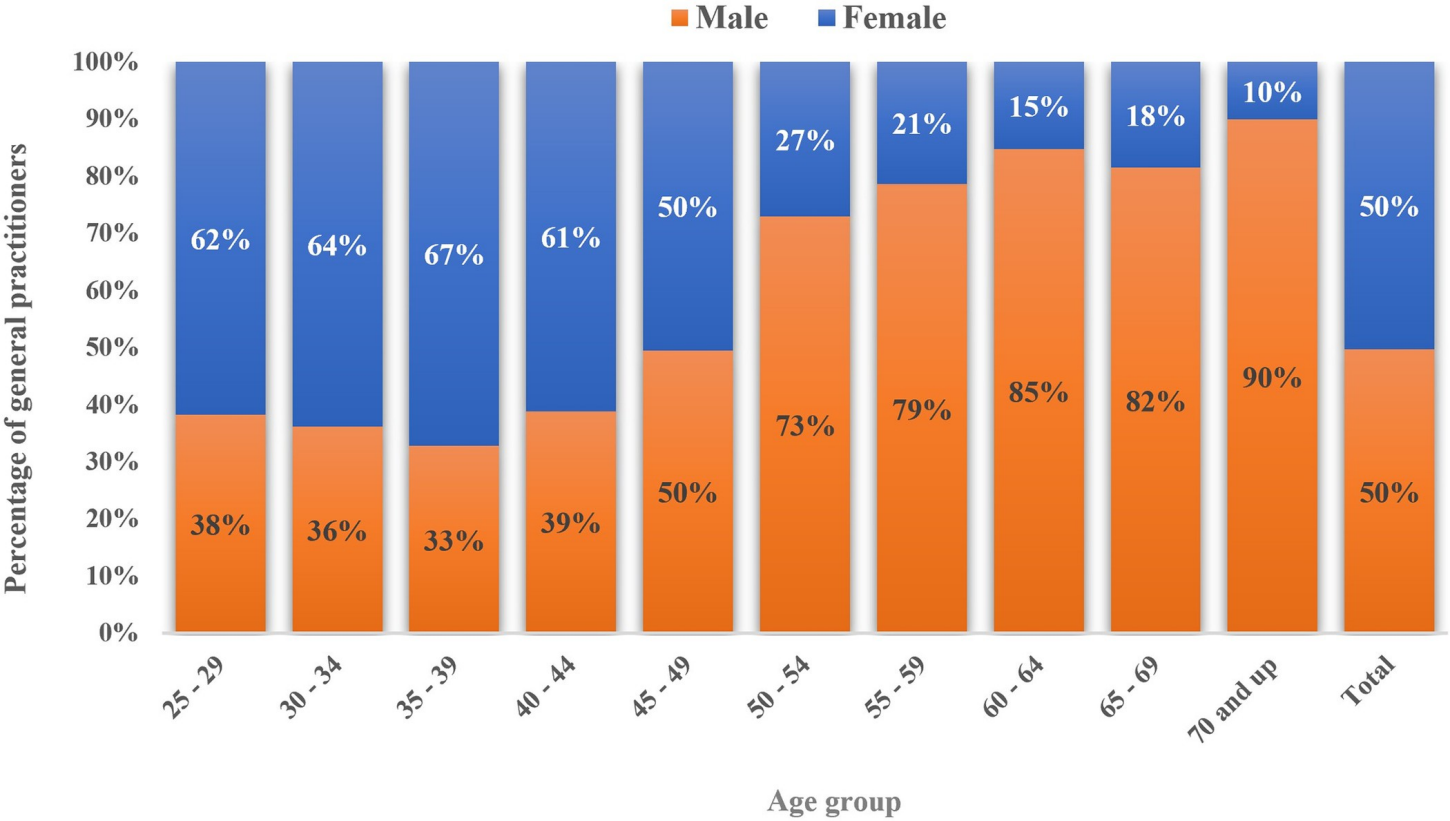
Gender distribution of GPs by age group.

**Fig 4:**
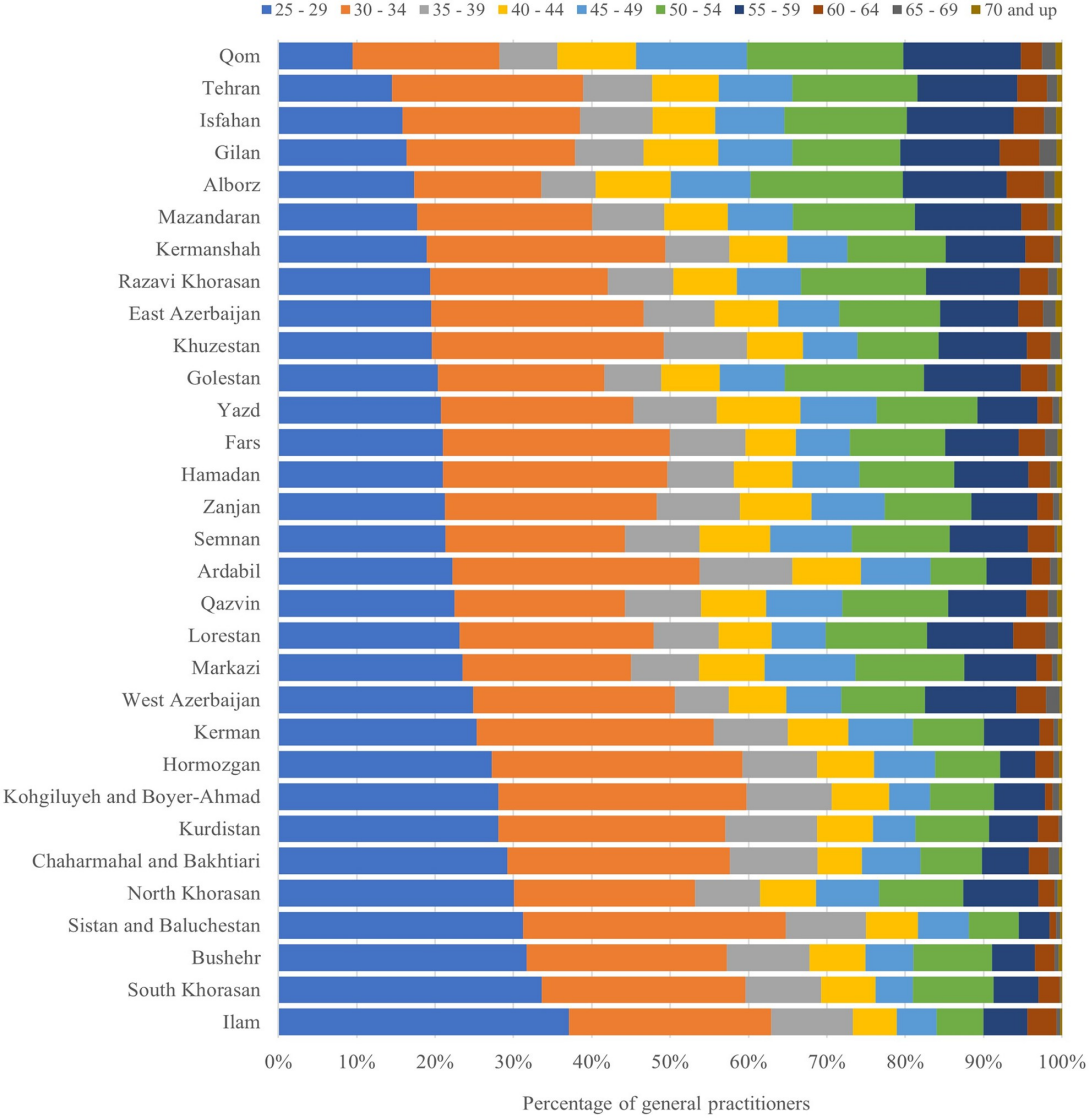
Distribution of active GPs by age group and province of practice.

The age distribution of the physicians according to sex is depicted in Fig 2, with the sex distribution within each age group shown in Fig 3. Moreover, Fig 4 illustrates the percentage distribution of active GPs across different age groups and the provinces where they practice medicine. Consequently, Qom Province had the lowest percentage of young GPs in relation to the overall number of active GPs in that province, while Ilam Province had the highest percentage.

**Fig 5:**
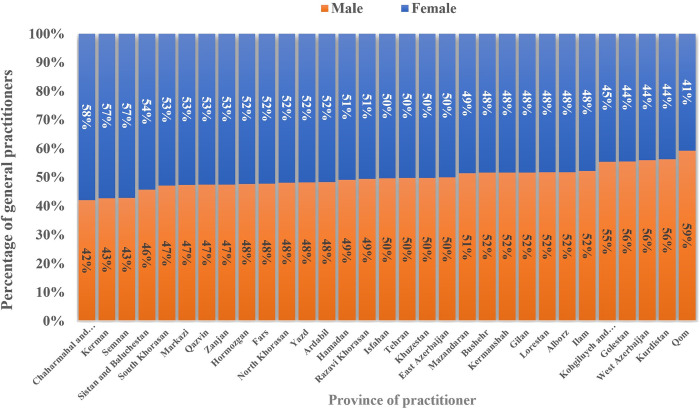
Gender distribution of GPs by province of practice.

Furthermore, Fig 5 delineates the gender distribution of active GPs across the provinces where they practice. Notably, Chaharmahal and Bakhtiari, and Qom Provinces had the highest and lowest percentages of female physicians among all the GPs within the same province.

**Fig 6:**
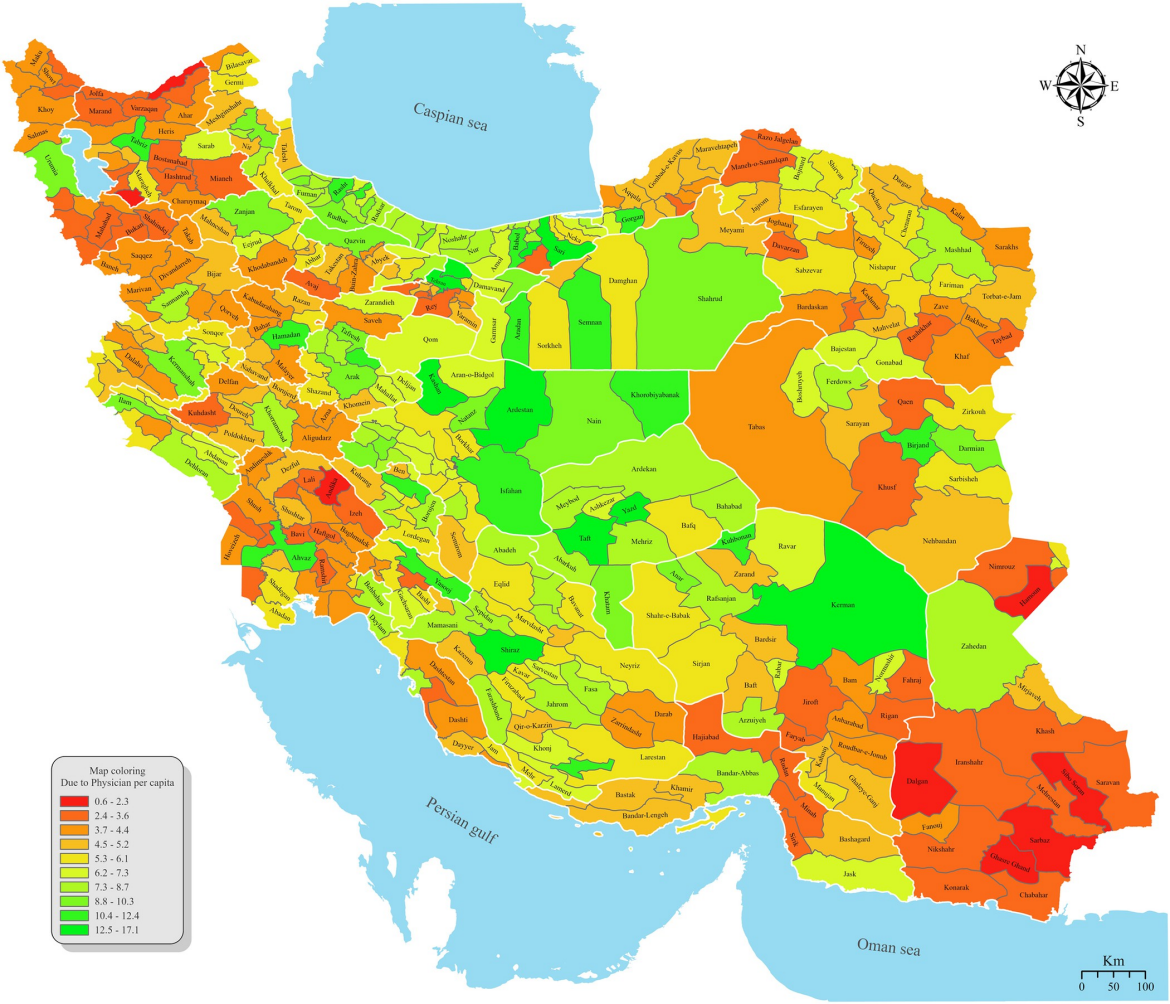
Intercity distribution map of active GPs per capita.

Fig 6 shows the city distribution map of active GPs in terms of physician per capita. A green city is associated with more GPs per capita. The base layer of the map used in Fig 6 is derived from the GADM database of Global Administrative Areas. The shapefile for Iran’s cities and provinces can be accessed directly at https://gadm.org/download_country.html. The terms of use for this data are available at https://gadm.org/license.html, and are compatible with the CC-BY 4.0 license.

**Fig 7:**
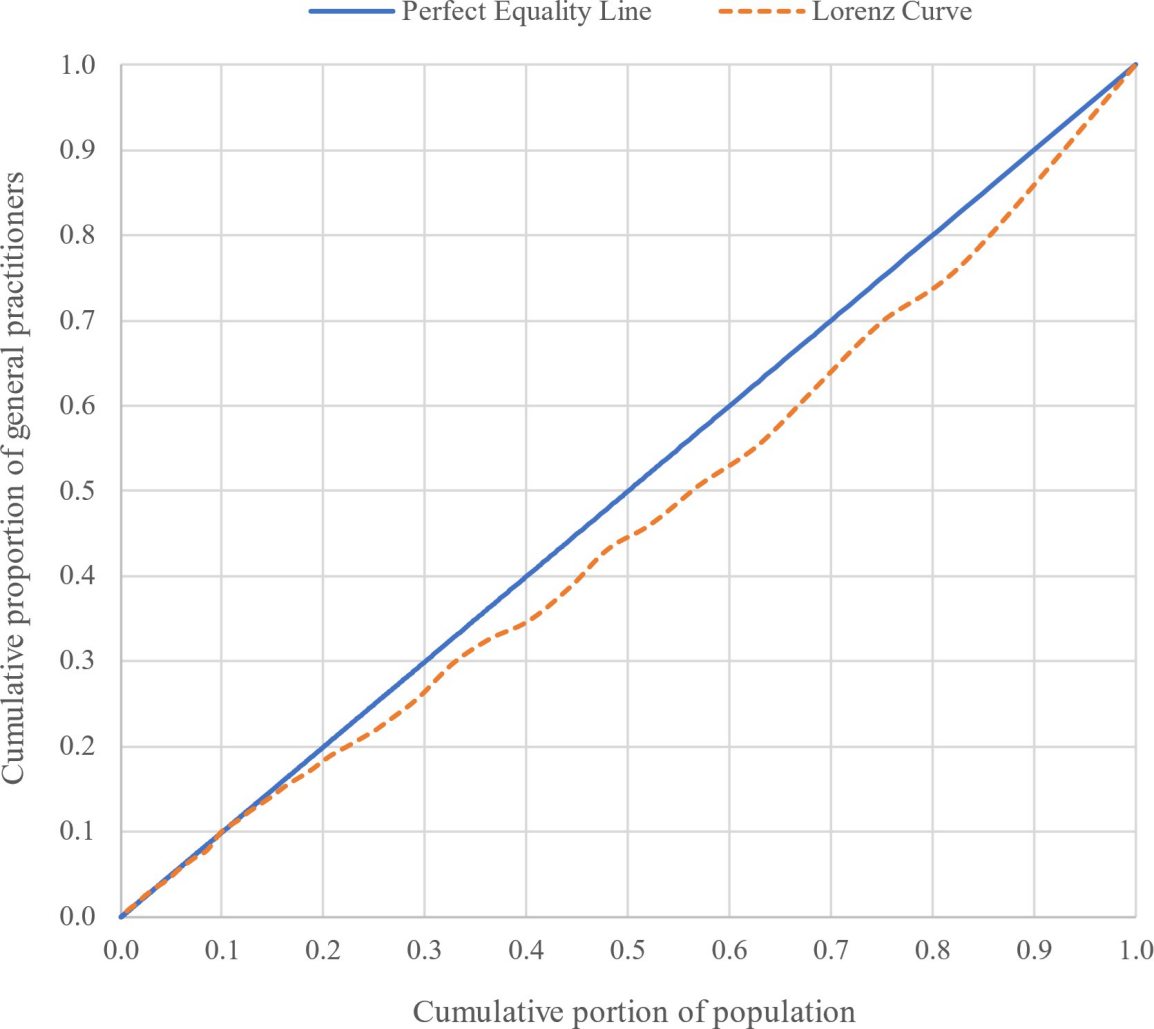
Lorenz curve and Gini coefficient distribution of GPs among provinces.

**Fig 8:**
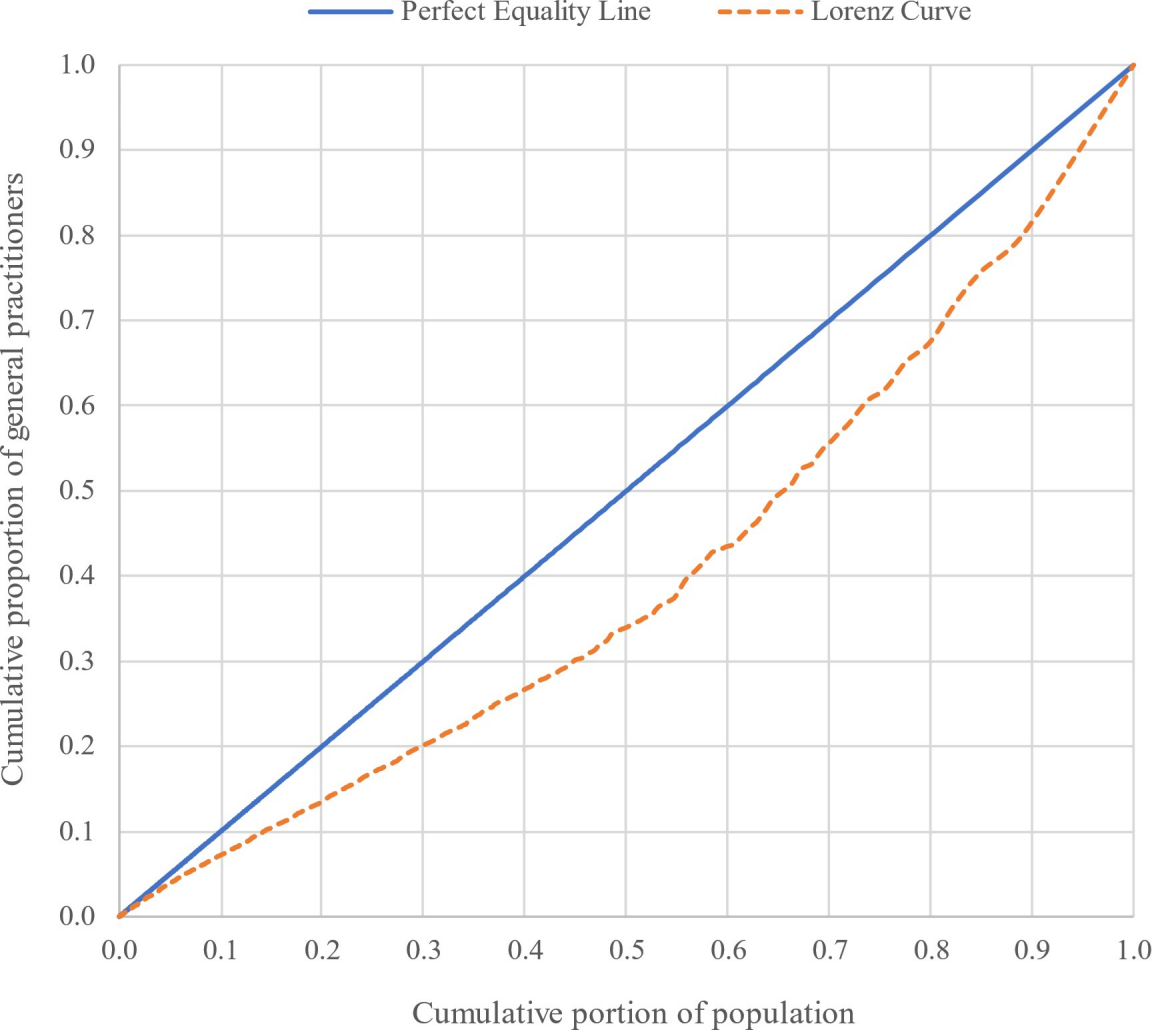
Lorenz curve and Gini coefficient distribution of GPs among cities.

Additionally, Figs 7 and 8 depict the interprovincial and intercity distributions of active GPs in relation to the Lorenz curve and the Gini coefficient, respectively. These graphical representations reveal that the Gini coefficient for the geographic distribution of GPs among provinces and cities equated to 0.08 and 0.20, respectively, signifying a fair and relatively fair geographic distribution of active GPs.

**Fig 9:**
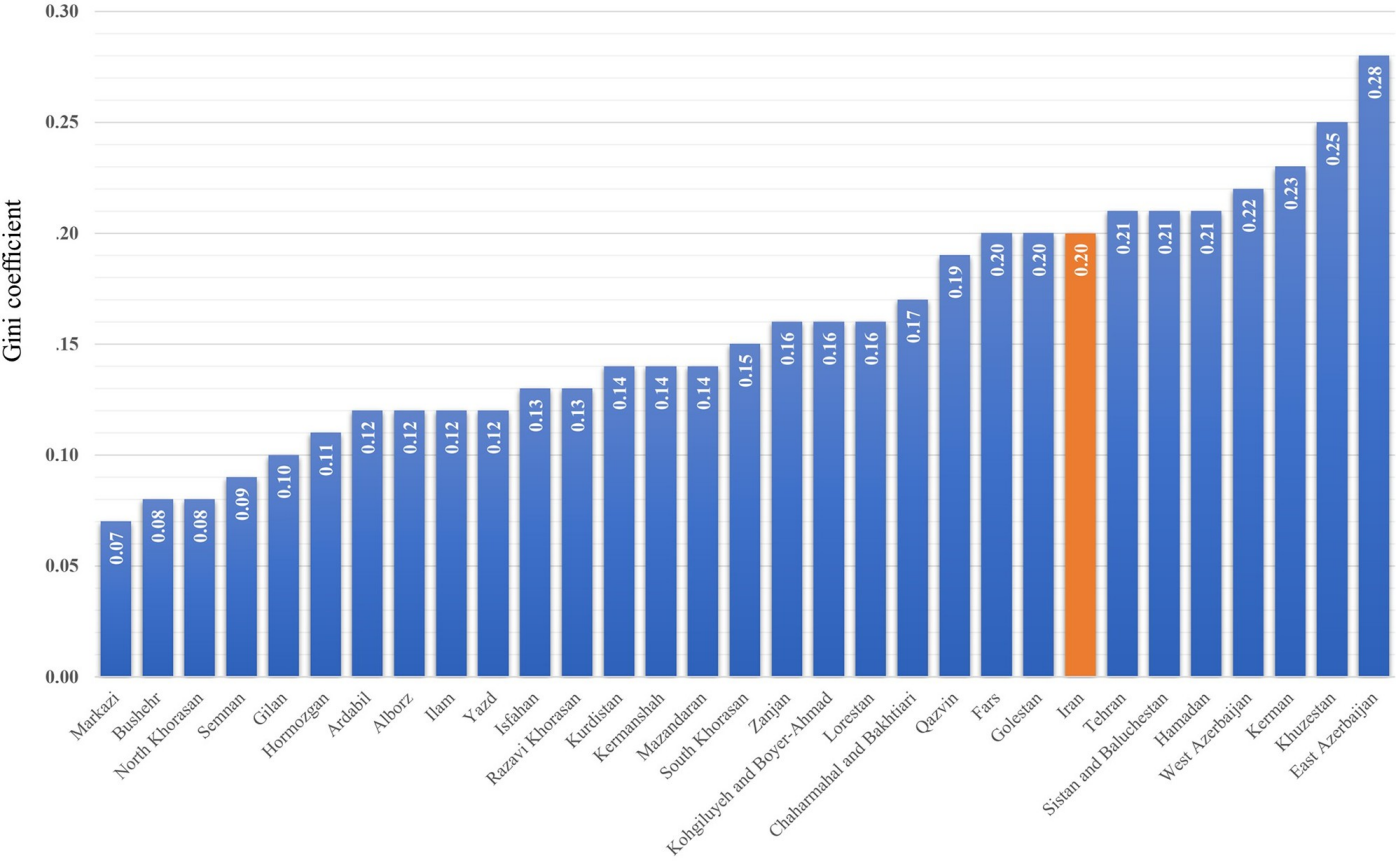
Gini coefficient distribution of GPs within each province.

In Fig 9, the Gini coefficients of the distribution of GPs across each province are shown in ascending order. According to this graphical representation, Central and East Azerbaijan provinces had the lowest and highest Gini coefficients, respectively, at 0.07 and 0.28, respectively.

## Discussion

The results indicated that in 2022, there were 67,852 active GPs who practice medicine for populations exceeding 85,874,000 individuals across 31 provinces of which 74% are urban, leading to an average of 7.9 active GPs per ten thousand people in the country. Notably, the provinces of Yazd, and Sistan and Baluchestan had the highest and lowest per capita numbers of GPs, with 11.6 and 4.3 practitioners per ten thousand people, respectively. In a study based on the data of 2016, there were 6.7 GPs per ten thousand population [27]. Considering the graduation of medical students in these years and the growth of the number of GPs in Iran, this ratio is consistent with the present study.

Countries that were more similar to Iran in terms of income group, population category, and economic status and information about the number of GPs were found were selected for comparison. Therefore, based on the latest available data, the number of GPs per ten thousand populations in Colombia (2021), Thailand (2020), Spain (2020), Mexico (2020), Turkey (2020), Saudi Arabia (2021), South Africa (2018), Poland (2017) and Egypt (2019) were 16.8, 9.3, 9.1, 8.9, 6.7, 6.0, 5.1, 4.1 and 3, respectively. Therefore, according to this index, Iran is in the middle of this list and above Turkey [28]. Nonetheless, comparing the quantity of GPs across countries is challenging due to variations in their classification methods. For instance, in the United States, general internists often fulfill a role like that of GPs in other countries but are categorized as medical specialists. Similarly, general pediatricians who provide care for children are considered specialists in many countries and are therefore not classified as GPs. In Iran, both groups of physicians (internal medicine and pediatrics) are regarded as specialists and are excluded from the statistical analysis.

Nationally, The per capita ratio of active GPs in the central areas of each province is more than other cities within the same province. In 2022, a cross-sectional study conducted in a Province of Iran had reached a similar result [29].

In terms of the age distribution of active GPs, results indicate a noticeable shift in the gender composition of physicians, with women representing more than 62% of physicians aged 25 to 34 years, reflecting a trend where women have come to outnumber men in this age group. Overall, the age groups 25 to 34 years and 50 to 59 years had the highest percentage of GPs. The decline in the number of GPs in the intervening age brackets may be attributed to factors such as diminished capacity for admitting medical students in the 1990s, a trend aligned with data on medical student enrollment published by the National Assessment Organization [30].

Given the escalating proportion of physicians older than 55 years, initiative-taking strategies are warranted to anticipate the retirement of physicians over the next decade. This issue is not unique to Iran, as member countries of the Organization for Economic Cooperation and Development (OECD) have grappled with similar challenges, prompting concerted efforts to augment the training capacity of GPs in response to the shortfall [31].

The research findings indicate the fairness of the distribution of GPs across provinces, with a Gini coefficient of 0.08. However, the distribution among cities within the country is relatively fair, as reflected by a Gini coefficient of 0.2. The difference in the number of units of analysis can significantly affect the values of the Gini Index. At the interprovincial level, with 31 units, the Gini Index may provide more general results, so that 0.08 was obtained for the distribution of GPs. However, at the intercity level, with 429 units, the Gini Index can offer more precise results regarding the inequality distribution, which was 0.2 in this study. And for the intraprovincial level, the results are shown in Fig 9. These differences indicate that analysis at different geographical levels can yield varying results and should be considered when interpreting the data.

Based on a cross-sectional study conducted in 2018 in South Khorasan [32], the Gini coefficient of the geographical distribution of GPs in this province was 0.38, while the results of our study showed this index to be 0.15. On the other hand, a cross-sectional study conducted in 2016 in Sistan and Baluchestan [33] estimated this intra-provincial Gini coefficient to be 0.03, while in our current study, it was 0.21. The reason for these discrepancies is that the data sources of those studies are only the data of governmental GPs published by the Iranian Statistics Center, while in our study all active GPs were calculated, whether governmental or non-governmental.

In 2016, a cross-sectional study conducted in Iran determined the Gini coefficient of the distribution of GPs in the public sector to be 0.17, signifying a fair distribution and aligning with the findings of the current study [34]. Similarly, another cross-sectional study in 2014 revealed a decreasing trend in the Gini coefficient of the distribution of GPs for the period 2010–2015, reaching 0.13 nationwide in 2010, indicating complete geographical equity in the allocation of these physicians, consistent with the present research [4]. Conversely, a 2015 cross-sectional study demonstrated a Gini coefficient of 0.21 for the intercity distribution of GPs in Kurdistan Province, whereas the present study reported a coefficient of 0.14, reflecting a slight variance from the current study’s results [35]. A descriptive study in 2016 investigating the Gini coefficient of GP distribution in Tehran Province found values ranging between 0.26 and 0.29 across different years. In contrast, the current study identified a Gini coefficient of 0.21 for the distribution of active GPs in Tehran Province, indicating a relatively fair distribution of physicians within the province [36]. Finally, a 2016 study in Khuzestan Province calculated the Gini coefficient of GP distribution to be 0.19, while the current study reported a coefficient of 0.25, potentially indicating a decline in equity in physician distribution within this province [37].

Internationally, a 2015 study in Turkey reported a Gini coefficient of 0.23 for the distribution of GPs [38], while studies in Greece and Albania revealed coefficients of 0.18 and 0.48, respectively [39]. In China, the estimated Gini coefficient of GPs distribution was 0.19 in 2019 [40], decreasing to 0.15 in 2021 [41].

An equitable distribution of physicians fosters more suitable geographic access for patients, which is a fundamental prerequisite for achieving universal health coverage. To ensure a balanced distribution of healthcare professionals in Iran, the government has implemented several policies, including the Family Physician Program and compulsory service in underserved areas for newly graduated medical professionals. These measures are designed to address regional disparities and improve access to healthcare in rural and less developed areas [42,43].

However, mere geographic access to healthcare providers is insufficient, as other factors, such as the quality of medical practitioners, standards of healthcare facilities, availability of medication and essential medical supplies, financial accessibility, and cultural congruence, also significantly impact the extent to which patients benefit from this human resource. It is imperative for governments to eliminate financial impediments to individuals’ utilization of medical services and facilitate adequate access to healthcare professionals who align with the cultural and ideological needs of society. Additionally, government subsidies in the healthcare sector should be directed toward those truly in need, thereby promoting fairness in accessing medical resources.

## Conclusion

The findings of the current study indicate that in 2022, there were 67,852 active GPs, leading to an average of 7.9 GPs per ten thousand people with a Gini coefficient of 0.08 for distribution. Women constitute 50% of active GPs. Notably, the provinces of Yazd, and Sistan and Baluchestan had the highest and lowest per capita numbers of GPs. Also, the results showed that there are always more active GPs per capita in the central areas of each province than in other cities within the same province. Moreover, Iran has a lower ratio of GPs per capita than countries with well-established healthcare systems, such as those in Europe. These results emphasize the need for policymakers to prioritize the implementation of appropriate strategies and interventions aimed at enhancing access to and equity in the geographical distribution of GPs between cities within each province. Furthermore, in light of the absence of a current and comprehensive database of active GPs and specialists in the country, it is imperative for the Ministry of Health and Medical Education, as the stewardship of the healthcare system, to establish and annually update this database. Such action will enable the assessment of changes in the distribution of physicians over specific time periods and facilitate the evaluation of physician redistribution policies within the country.

### Policy implications of the study

The results of this study showed that in general, the distribution of GPs in Iran is fair; But the distribution within each province shows that there are more differences in the distribution of GPs within the province, the causes of which should be investigated by policymakers in each province separately. Also, the number of active GPs in each city was determined and displayed as a map, which can be cited and used for the allocation of resources for the managers of each of these regions. Furthermore, to anticipate the retirement of physicians over the next ten years, initiative-taking strategies are required in light of the growing proportion of physicians over 55 years old, particularly in provinces where a higher percentage of GPs will soon reach retirement age.

### Limitations of the study

In the field of distribution of GPs in Iran, the main source of data for the studies that have been carried out thus far is GPs (according to the data of the Iranian Statistics Center and the Ministry of Health and Medical Education) or all physicians with a medical system number (active and inactive). The distribution of active GPs who were in the governmental or nongovernmental sector was calculated. Therefore, due to this difference in data sources, it is not possible to fully compare the results with the results of the studies conducted thus far. Therefore, this issue should be considered when comparing the results of this research with those of other domestic studies.

### Suggestions for researchers

The current study provides a description of the geographical distribution of active GPs in Iran with respect to age and gender at both the provincial and city levels. However, to offer a more comprehensive solution for policymakers, it is imperative to interpret the research findings based on methodologies for assessing shortages or surpluses of human resources, per capita adjustments according to need (need-adjusted index), and comparisons with similar countries worldwide. Therefore, it is recommended that studies with the stated objectives be conducted by other researchers, building upon the findings of the present study.

## Supporting information

S1 Data (XLSX)
